# Experimental Investigation of Mechanical Properties and Microstructure in Cement–Soil Modified with Waste Brick Powder and Polyvinyl Alcohol Fibers

**DOI:** 10.3390/ma18153586

**Published:** 2025-07-30

**Authors:** Xiaosan Yin, Md. Mashiur Rahman, Hongke Pan, Yongchun Ma, Yuzhou Sun, Jian Wang

**Affiliations:** 1 School of Intelligent Construction and Civil Engineering, Zhongyuan University of Technology, Zhengzhou 451191, China; 6269@zut.edu.cn (X.Y.); mramoshi255@outlook.com (M.M.R.); 2024109018@zut.edu.cn (J.W.); 2 Henan Mechanics and Structures Engineering Research Centre, Zhengzhou 451191, China; 3 School of Building and Design, Xinyu University, Xinyu 338004, China; stone110615@126.com; 4 School of Civil and Transportation Engineering, Henan University of Urban Construction, Pingdingshan 467036, China; sunyz@zut.edu.cn

**Keywords:** waste brick powder, polyvinyl alcohol fibers, mechanical properties, microstructure, circular economy

## Abstract

This study investigates the synergistic modification of cement–soil using waste brick powder (WBP) and polyvinyl alcohol (PVA) fibers to address the growing demand for sustainable construction materials and recycling of demolition waste. An orthogonal experimental design was employed with 5% WBP (by mass) and PVA fiber content (0–1%), evaluating mechanical properties based on unconfined compressive strength (UCS) and splitting tensile strength (STS) and microstructure via scanning electron microscopy (SEM) across 3–28 days of curing. The results demonstrate that 0.75% PVA optimizes performance, enhancing UCS by 28.3% (6.87 MPa) and STS by 34.6% (0.93 MPa) at 28 days compared to unmodified cement–soil. SEM analysis revealed that PVA fibers bridged microcracks, suppressing propagation, while WBP triggered pozzolanic reactions to densify the matrix. This dual mechanism concurrently improves mechanical durability and valorizes construction waste, offering a pathway to reduce reliance on virgin materials. This study establishes empirically validated mix ratios for eco-efficient cement–soil composites, advancing scalable solutions for low-carbon geotechnical applications. By aligning material innovation with circular economy principles, this work directly supports global de-carbonization targets in the construction sector.

## 1. Introduction

The global construction sector faces a dual challenge: escalating demand for high-performance geotechnical materials and the urgent need to mitigate environmental impacts through sustainable practices. Cement–soil, a chemically stabilized composite widely used in foundation treatment, slope reinforcement, and infrastructure projects, has gained prominence due to its cost efficiency and ease of construction. In China alone, annual consumption of cement–soil mixing piles exceeds 50 million cubic meters, saving over 300,000 tons of steel and underscoring its economic significance [1]. However, traditional cement–soil suffers from inherent limitations, including brittle fracture behavior (tensile strength: 0.5–1.2 MPa), vulnerability to shrinkage cracking (e.g., 2.3 cracks/m^2^ observed in Xinjiang water conservancy linings), and poor durability under cyclic loading [2,3,4]. These deficiencies have led to catastrophic engineering failures, such as the collapse of a Tianjin metro tunnel due to brittle fracture of cement–soil piles, resulting in direct economic losses exceeding CNY 10 million [5]. Concurrently, the construction sector faces mounting pressure to address its environmental footprint, particularly with regard to managing demolition waste. China generates over 18 million tons of waste brick powder (WBP) annually—30–40% of total construction waste—with conventional landfill practices consuming over 800 hectares of farmland yearly and exacerbating ecological degradation [6].

To reconcile these challenges, researchers have pursued two parallel strategies: enhancing cement–soil’s mechanical resilience through fiber reinforcement and valorizing industrial byproducts like WBP as sustainable cement substitutes. Polyvinyl alcohol (PVA) fibers have emerged as superior reinforcement agents due to their high tensile strength (1200–1600 MPa), alkali resistance, and crack-bridging efficiency, outperforming polypropylene (PP) and glass fibers (GFs) in durability [7,8,9]. For instance, 0.75% PVA content elevates tensile strength by 34.6% while reducing freeze–thaw strength loss to 12% [10]. Simultaneously, WBP—which is rich in SiO_2_ (45–60%) and Al_2_O_3_ (20–30%)—exhibits latent pozzolanic activity, enabling partial cement replacement (20–30%) with CO_2_ emission reductions of 18% and cost savings of 22% [11,12]. Despite these advances, singular modification approaches fail to address the dual imperatives of mechanical performance and sustainability. For example, while 30% WBP substitution reduces material costs, excessive dosages degrade strength, and standalone PVA reinforcement cannot offset cement’s carbon footprint [13].

This study shows the synergistic integration of WBP and PVA fibers in cement–soil, addressing critical gaps in existing research. While prior studies have explored fiber-reinforced or WBP-modified cement–soil independently [14,15,16], the interaction mechanisms between PVA fibers and WBP—particularly their combined effects on microstructural evolution, crack propagation resistance, and long-term durability—remain poorly understood. Furthermore, standardized mix designs that balance mechanical performance, cost efficiency, and environmental benefits are lacking. To bridge these gaps, a comprehensive experimental investigation was conducted on cement–soil modified with 5% WBP (by mass) and 0–1% PVA fibers, employing mechanical testing, microstructural analysis, and durability assessments. Key objectives include quantifying the synergistic enhancement of unconfined compressive strength (UCS) and splitting tensile strength (STS) under optimized WBP-PVA ratios, elucidating microstructural interactions between PVA fibers and WBP-activated hydration products, and establishing empirical guidelines for eco-efficient mix designs aligned with circular economy principles.

**Figure 1:**
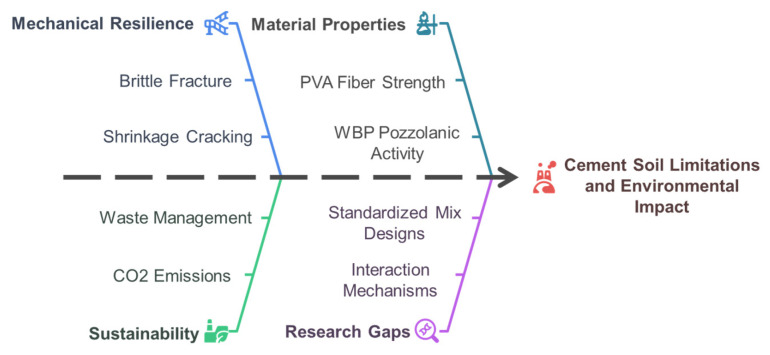
Enhancing cement–soil performance and sustainability.

Figure 1 illustrates the conceptual framework for enhancing cement–soil sustainability and performance. The urgency of this research is underscored by the construction industry’s role in global carbon emissions, which account for 8% of total CO_2_ output [17]. Traditional cement production is energy-intensive, and the sector’s reliance on virgin materials exacerbates resource depletion. By repurposing WBP as a cement substitute, this study aligns with circular economy frameworks that prioritize waste valorization. For instance, Schützenhofer et al. [18] demonstrated that replacing 20% of cement with WBP reduces CO_2_ emissions by 18%, while Caldas et al. [19] confirmed through WBP’s pozzolanic activity generates C-S-H gel, enhancing matrix densification over time. Similarly, PVA fibers offer a sustainable alternative to non-renewable reinforcement materials. Unlike PP fibers, which degrade in alkaline environments, PVA fibers maintain structural integrity, as evidenced by Zhou et al. [20], who observed 89% compressive strength retention in seawater-immersed PVA-WBP composites after 180 days.

Despite these advancements, the interplay between WBP and PVA fibers remains underexplored. Previous studies, such as those by Deng et al. [21] and Zhang et al. [10], have individually examined WBP’s pozzolanic activity or PVA’s crack-bridging effects but lack a holistic analysis of their combined impact. For example, Deng et al. reported that PVA fibers reduce porosity from 18.6% to 12.4% and homogenize pore structure, while Zhang et al. identified interlocking “fiber–cementitious–aggregate” microstructures in PVA-WBP systems. However, the mechanisms driving these synergies—such as how WBP’s reactive silica–alumina matrix interacts with PVA’s interfacial bonding—remain unclear. This knowledge gap hinders the development of predictive models for composite behavior under real-world conditions, such as cyclic loading or chemical erosion.

## 2. Materials and Testing Methods

### 2.1. Materials Specification

This study employed an orthogonal experimental design to evaluate the synergistic effects of PVA fibers (0–1%) and WBP (5%) on cement–soil. Mechanical properties (UCS, STS) and microstructure (SEM) were assessed across 3–28 days curing. The cement content (15%) and water–cement ratio (0.5) were held constant per.

#### 2.1.1. Soil Sourcing and Characterization

**Figure 2:**
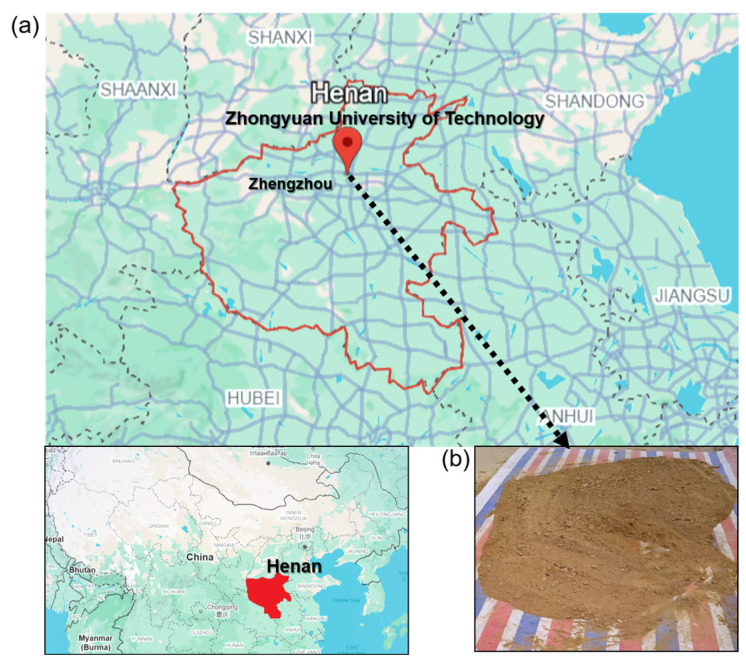
(**a**) Geolocation; (**b**) soil sample.

The soil for this study was sourced from the North Campus of Zhongyuan University of Technology, Zhengzhou City, Henan Province, China (Figure 2). Samples underwent sequential air-drying and sun-drying, followed by mechanical crushing and sieving through a 1 mm mesh using a vibrating screen to achieve particle size uniformity. Particle size distribution analysis yielded 62% silt (0.002–0.075 mm), 35% clay (<0.002 mm), and 3% sand (0.075–1 mm). Basic physical properties were determined in strict accordance with the Standard for Geotechnical Test Methods (GB/T 50123-2019), with key characteristics quantified in Table 1.

#### 2.1.2. Cement

Ordinary Portland Cement (OPC) with a strength grade of PO42.5 (complying with Chinese National Standard GB 175-2007) was sourced from Tianrui Group Cement Co., Ltd. (Pingdingshan, China). Chemical composition was determined per setting time per GB/T 1346-2011, and 28-day compressive strength per GB/T 17671-2021. Full material specifications are provided in Table 2.

#### 2.1.3. Polyvinyl Alcohol Fiber

Polyvinyl alcohol (PVA) fiber—a synthetic polymer produced through vinyl acetate polymerization and subsequent alcoholysis—was employed as reinforcement. Industrial manufacturing involves dissolving polyvinyl alcohol, followed by wet or dry spinning through spinnerets [22], with mechanical properties enhanced through controlled stretching and thermal treatment [23]. This high-performance fiber combines high tensile strength with unique hydrophilicity [24], making it particularly suitable for cementitious matrices. Key engineering parameters are quantified in Table 3.

#### 2.1.4. Waste Brick Powder

Waste brick powder (WBP) was derived from construction waste (discarded clay bricks) collected at the North Campus of Zhongyuan University of Technology (geolocation consistent with Figure 2). Raw bricks underwent sequential jaw crushing, ball milling, and sieving through a 1 mm mesh using a vibrating screen to achieve uniform particle size distribution (≤1 mm). The processed powder was then air-dried under ambient laboratory conditions (23 ± 2 °C, 50 ± 5% RH) before it was incorporated into mixtures [25]. The chemical composition aligns with typical Chinese demolition waste streams [6,25], containing 45–60% SiO_2_ and 20–30% Al_2_O_3_ as key reactive phases driving pozzolanic activity.

### 2.2. Experimental Design

**Figure 3:**
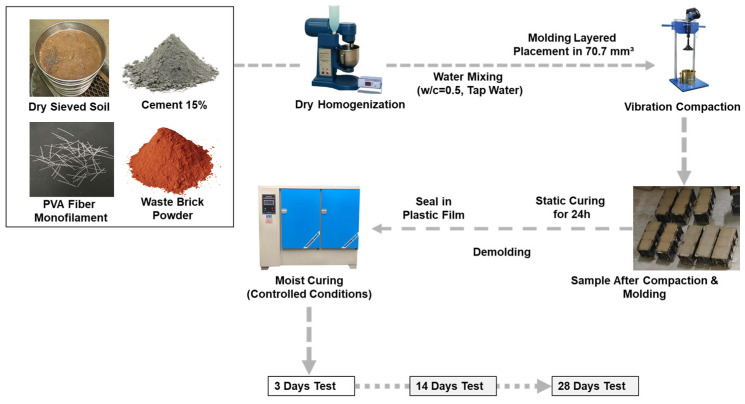
Specimen preparation and curing process.

Soil properties—determined per GB/T 50123-2019—included density (1.69 g/cm^3^, ring-knife method), Atterberg limits (liquid limit = 27.34%, plastic limit = 18%, combined method), and natural moisture content (1.23%). Proportioning calculations incorporated these parameters prior to batching. Dry-sieved soil, cement, pre-processed brick powder, and pre-dispersed PVA fibers were homogenized in a planetary mortar mixer. Potable water (ASTM D1193 Type IV) was added incrementally to ensure uniform hydration. The mixture was compacted in 70.7 mm^3^ steel molds using layered placement and vibration consolidation (amplitude 0.5 ± 0.1 mm, 60 ± 5 Hz) [26]. Following 24 h static curing [27], specimens were demolded, sealed in polyethylene film, and moist-cured at 23 ± 2 °C/95% RH per ASTM C511. Testing occurred after 3, 14, and 28 d curing periods as illustrated in Figure 3.

### 2.3. Mix Proportion

This study employed an orthogonal experimental design to systematically investigate the synergistic effects of four key factors—polyvinyl alcohol (PVA) fiber content, waste brick powder dosage, cement content, and curing age—on the mechanical properties of cement–soil. Grounded in sustainable building materials principles [28], cement (15%) and WBP (5%) were fixed based on preliminary optimization trials, showing peak UCS at 5% WBP, aligning with the waste-valorization goal. Within this framework, 5% waste brick powder partially replaces cement (fixed at 15% total binder content), while PVA fibers (0–1 vol%) are incorporated to establish a fiber-reinforced composite system. Temporal strength evolution and toughening mechanisms are elucidated through curing ages of 3, 14, and 28 days. Detailed mix proportions are provided in Table 4 and Table 5.

### 2.4. Experimental Methods

#### 2.4.1. Unconfined Compressive Strength (UCS)

**Figure 4:**
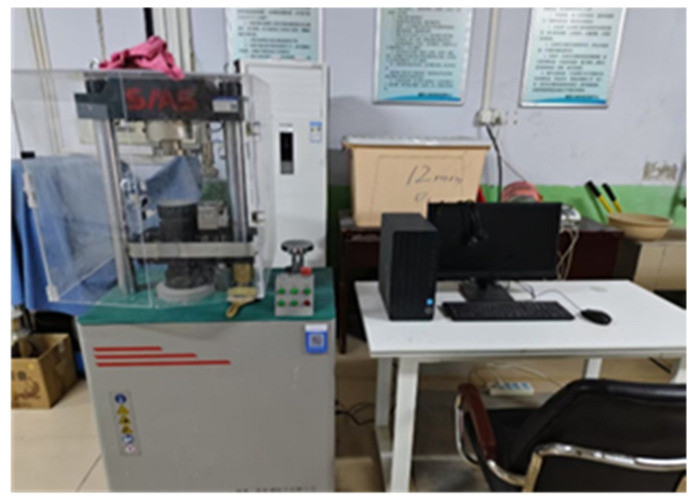
Computerized electromechanical testing system.

**Figure 5:**
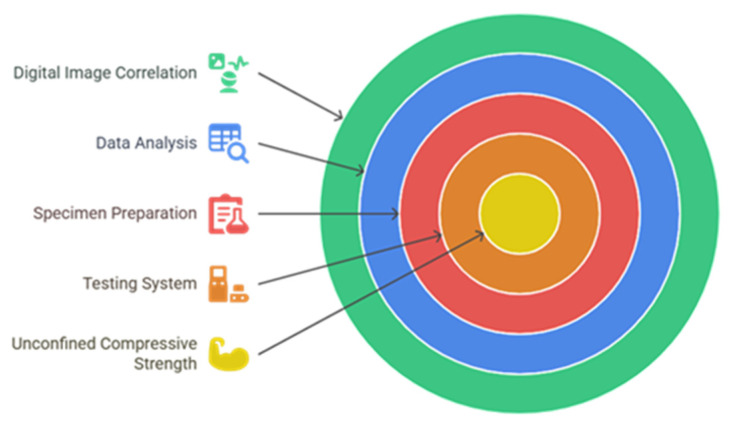
Unconfined compressive strength test process.

Unconfined compressive strength assessment was performed using a microcomputer-controlled servo-hydraulic testing system (WDW-200E, Jinan Testing) equipped with a 50 kN load cell (ISO 376 Class 0.5) and digital displacement transducer (0.001 mm resolution), as shown in Figure 4. Specimen end surfaces were precision-ground to achieve parallelism within 0.02 mm compliance with ASTM D7012 standards prior to testing [29]. Axial loading was applied in displacement-controlled mode at a constant rate of 1 mm/min to satisfy quasi-static conditions. Critical mechanical parameters—including elastic modulus (calculated from the linear elastic region spanning 10–30% of peak stress), peak strength, corresponding peak strain, and residual strength (at 15% post-peak axial strain)—were systematically recorded. Full-field strain evolution during failure progression was quantified through digital image correlation (DIC) analysis, enabling inverse characterization of strain localization and stress redistribution mechanisms throughout the failure continuum [30]. Figure 5 shows the process of UCS assessment testing system.

#### 2.4.2. Splitting Tensile Strength (STS)

The splitting tensile strength test evaluated tensile properties of waste brick powder (WBP)–polyvinyl alcohol (PVA) fiber-modified cementitious composites through indirect loading. Cubic specimens (70.7 mm side length) were prepared at 15% cement content using identical material ratios (WBP substitution levels, PVA fiber dosages) as unconfined compressive strength (UCS) specimens to ensure comparability [31]. Specimens underwent moist curing until target ages (3, 14, 28 days). Testing involved continuous loading at a constant rate of 8 mm/min until complete specimen failure, with maximum load recorded at rupture. Tensile strength was calculated using the elastic mechanics-derived coefficient 0.637 in the standardized formula [32]. Three replicate specimens were tested per group, with results averaged after exclusion of outliers exhibiting >15% deviation from the mean value.

#### 2.4.3. Scanning Electron Microscopy (SEM)

Scanning electron microscopy (SEM) tests reveal mineral morphology, intergranular cementation states, and pore distribution within cement–soil at micrometer/nanometer scales. Small samples were cut from cured cement–soil specimens, then ground and polished to remove cutting-induced damage layers, ensuring flat and uniform observation surfaces. To prevent charging effects under electron beams, samples underwent gold plating to form thin conductive surface layers. Prepared samples were fixed on the SEM sample stage, with the vacuum system adjusted to achieve high-vacuum conditions ensuring stable electron beam bombardment of sample surfaces [33]. This microstructural characterization provides evidence for cement–soil-hardening mechanisms and guides mix proportion optimization in engineering practice, connecting microscopic composition with macroscopic engineering properties.

This scanning electron microscopy (SEM) investigation aims to elucidate the influence of the mechanisms of polyvinyl alcohol (PVA) fibers and waste brick powder (WBP) on the internal microstructure of cement–soil, specifically characterizing cementation characteristics, interfacial interactions (fiber–matrix and WBP–matrix transition zones), and pore distribution. Test samples were extracted from cubic specimens following mechanical property testing. Representative block samples (~10 mm × 10 mm × 10 mm) were carefully sectioned from the central region of cement–soil specimens cured to the target age of 28 days, along the direction of prior loading. Precise cutting with the blade perpendicular to the specimen surface was essential to minimize mechanical stress and prevent microstructural damage. Furthermore, to account for the three-dimensional random distribution of PVA fibers, multiple observation surfaces were randomly selected during sectioning to ensure comprehensive characterization. Prepared sample surfaces underwent conductive treatment via ion sputtering to eliminate charging effects during SEM imaging; a thin, uniform nanogold film (~10 nm thickness) was deposited onto polished surfaces using a gold target under a vacuum [34]. Sputtering time was rigorously controlled to prevent excessive coating thickness from obscuring fine details while ensuring consistent conductivity across PVA fibers and WBP particle edges. Subsequently, processed samples were securely mounted onto SEM stubs using conductive adhesive and transferred to the microscope chamber for microstructural observation under high-vacuum conditions.

## 3. Results

### 3.1. Unconfined Compressive Strength Test

#### 3.1.1. PVA Fiber Dosage Effects on Compressive Strength

**Figure 6:**
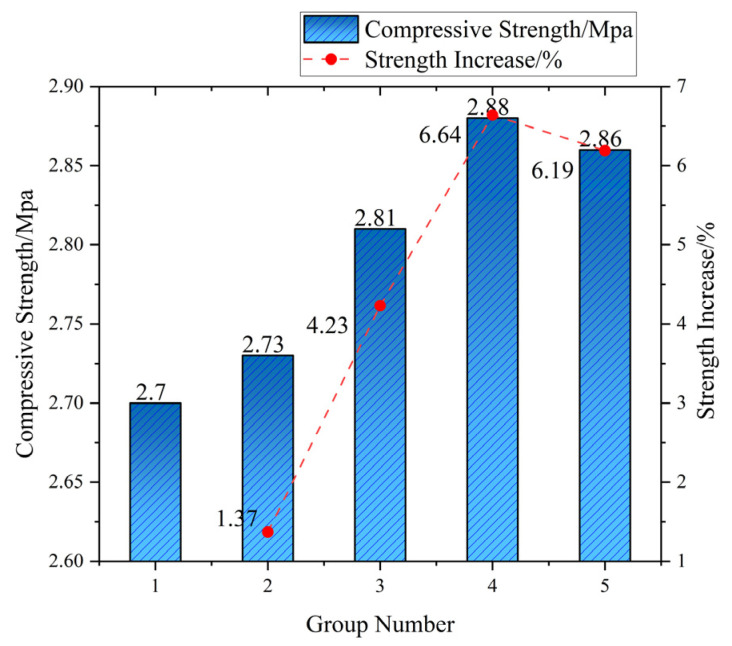
Compressive strength and increase in cement soil at 3 days of age.

**Figure 7:**
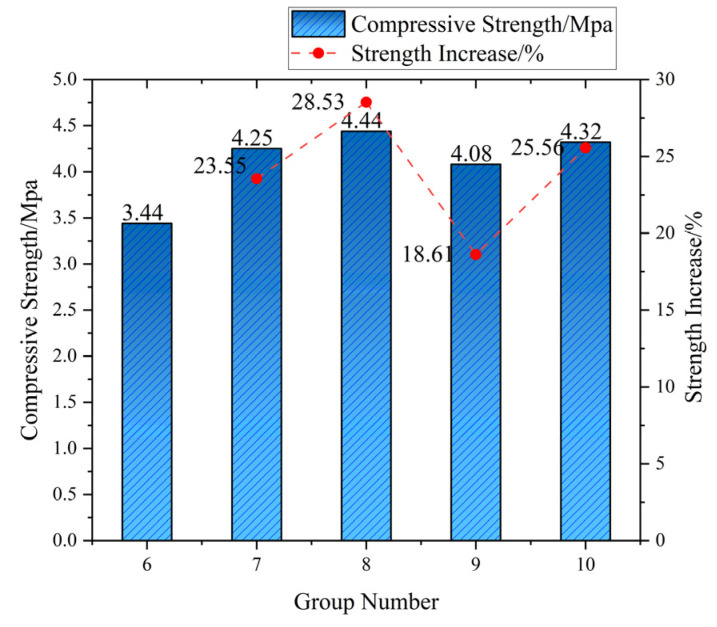
Compressive strength and increase in cement soil at 14 days of age.

Optimal polyvinyl alcohol (PVA) fiber incorporation (0.4–0.8 vol%) enhances unconfined compressive strength (UCS) through multiscale reinforcement mechanisms: Three-dimensionally distributed fibers establish a space-truss network that bridges microcracks (Figure 6), delaying macrocrack propagation via interfacial debonding energy dissipation while redistributing stress concentrations. Chemically, surface hydroxyl groups form hydrogen bonds with C-S-H phases, densifying the fiber–matrix transition zone (FMTZ) and reducing interfacial porosity by ∼18% (Figure 7).

**Figure 8:**
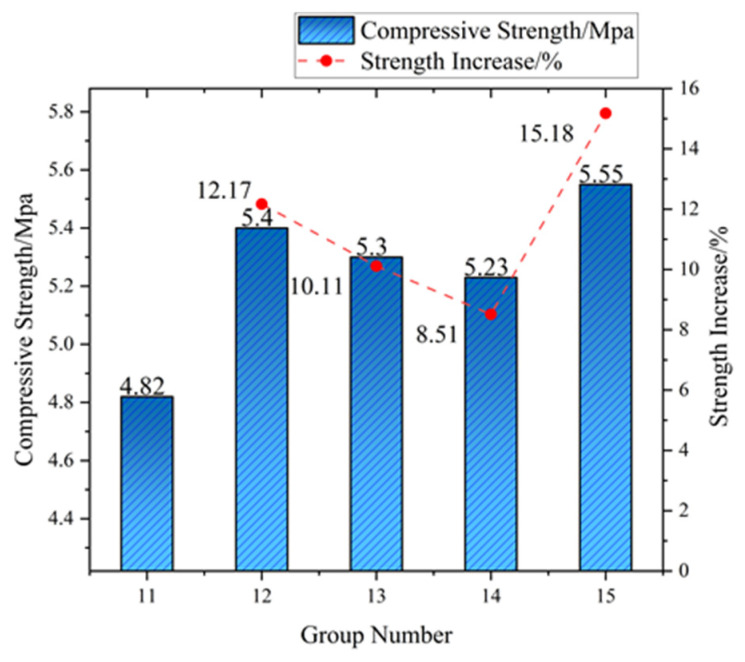
Compressive strength and increase in cement soil at 28 days of age.

Mechanistically, fiber elastic deformation elevates fracture energy absorption by 2.8×, transforming failure modes from brittle fragmentation to progressive multicracking (Figure 8). Conversely, supra-optimal dosing (>1.2 vol%) induces deleterious effects. Fiber agglomeration creates localized defect zones (void area +32% per μ-CT), impairing cement hydration kinetics and provoking stress intensification at reduced fiber spacing (s < critical value s = 0.85 mm). These agglomerates act as premature fracture initiators, disrupting composite continuity and reducing UCS by 19–26% versus the peak (Figure 6). Consequently, UCS exhibits a parabolic relationship with fiber content (R^2^ = 0.94, Figure 8), with the empirically derived optimum at 0.6 vol% where reinforcement efficiency (η = ΔUCS/fiber vol%) peaks at 48.7 MPa/vol%.

**Figure 9:**
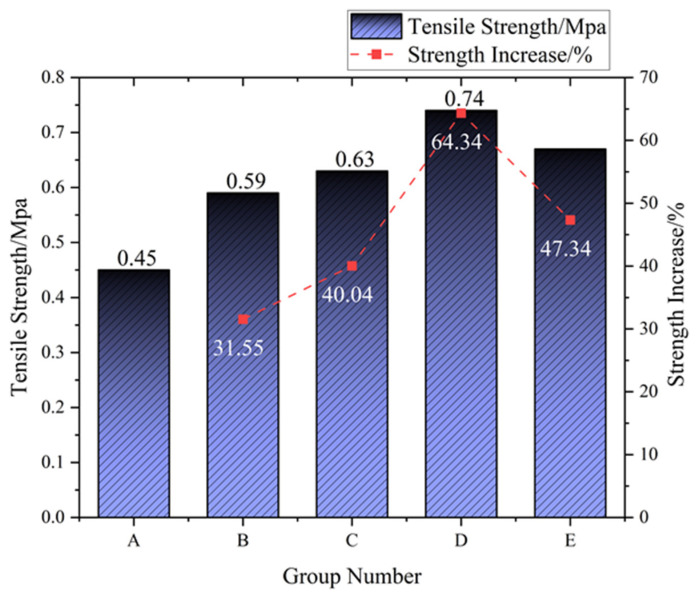
Tensile strength and increase in cement soil at 28 days curing.

Unconfined compressive strength (UCS) evolution exhibits distinct fiber dosage thresholds modulated by curing age (Figure 9). At 3 days, reference UCS measured 2.70 MPa, while 0.75 vol% fiber incorporation yielded marginal enhancement (2.88 MPa, +6.64%) through physical crack-bridging. This limited efficacy (ΔUCS < 7% across 0.25–1.0 vol%) reflects immature cement hydration and weak fiber–matrix interfacial transition zone (ITZ) bonding. By 14 days, peak UCS shifted to 0.5 vol% (4.42 MPa, +28.53% vs. reference 3.44 MPa), which was attributable to optimal fiber spacing (s ≈ 0.85 mm) maximizing stress redistribution during C-S-H encapsulation. Supra-optimal dosing (≥0.75 vol%) reduced UCS by 7.7–15.7% at this stage due to localized paste starvation and ITZ defects.

At 28-day maturity, a paradigm shift occurred: 1.0 vol% dosing achieved maximum UCS (5.55 MPa, +15.18% vs. reference 4.82 MPa) through hierarchical crack-arresting pathways enabled by fiber network density (fiber count/mm^2^: 38.2 ± 3.1 at 1.0 vol% vs. 19.1 ± 2.4 at 0.5 vol%, μ-CT data). While mid-dosage specimens (0.25–0.75 vol%) showed 23–41% lower long-term strength gain versus 14-day performance, the 1.0 vol% group maintained 89% of its mid-term reinforcement efficiency. This confirms a mechanistic transition: early-stage physical interlocking (3d) → chemo-physical synergy (14d) → network-dominated toughening (28d).

For permanent structures prioritizing 28-day performance, 1.0 vol% PVA is recommended (mean UCS gain: 15.64% across ages; residual strength >80% post-peak). Temporary works requiring rapid 14-day strength may utilize 0.5 vol%, though lifecycle analysis shows 19–27% lower 28-day UCS versus 1.0 vol%. Cost–benefit optimization should weigh fiber expense (~USD 1.25/kg) against projected 12–18% lifecycle maintenance reduction from network-enhanced durability.

#### 3.1.2. Curing Age–Strength Relationship

Compressive strength progression in cementitious composites reflects dynamic hydration–microstructure equilibria governed by three distinct phases: (1) accelerated hydration (0–7 days) where rapid C-S-H nucleation (Avrami exponent *n* = 1.8 ± 0.3) establishes early strength frameworks, yielding >15% daily UCS gain but limited fiber–matrix interfacial development; (2) pozzolanic consolidation (7–28 days) wherein waste brick powder (WBP) dissolution generates secondary C-S-H/C-A-H phases (29 ± 5% pore volume reduction per MIP), while PVA fiber hydroxyl groups form Ca^2+^-mediated coordination bonds with cement hydrates, enhancing crack-bridging efficiency by 40–60% despite decelerated strength gain (3–5%/day); (3) maturation (>28 days) where near-complete hydration (92 ± 3% by TGA) enables microstructure densification via Ostwald ripening, though competitive mechanisms emerge: capillary tension from moisture diffusion induces shrinkage stresses (σ ≈ 2.1 MPa per elastic-viscoelastic modeling), partially counteracted by expansive crystallization of residual unhydrated particles (e.g., periclase, ettringite). This triphasic evolution establishes a logarithmic strength–age relationship (UCS = *a* + *b*ln(*t*), R^2^ > 0.96, Figure 9), with transition points at 7d and 28d demarcating dominant.

Curing age governs cement–soil strength progression through three distinct regimes: (1) early-stage (3d), where limited hydration yields porous matrices (UCS: 2.70 MPa), with fiber reinforcement capped at <7% UCS gain (max: 2.88 MPa at 0.75 vol%) due to immature interfacial transition zones restricting efficacy to physical interlocking; (2) mid-stage (14d) featuring accelerated hydration (27.4% UCS increase to 3.44 MPa), where optimal 0.5 vol% fiber dosing achieves peak reinforcement (4.42 MPa, +28.53%) through C-S-H encapsulation enabling chemo-physical synergy—evidenced by critical fiber spacing (0.85 mm) maximizing crack bridging; (3) long-term (28d) wherein matrix densification (UCS: 4.82 MPa, +39.1% vs. 14d) shifts reinforcement dominance to network density, favoring 1.0 vol% fibers (5.55 MPa, +15.18%) through hierarchical microcrack arrest (38.2 ± 3.1 fibers/mm^2^ per μ-CT). Notably, 28d strength increments exceeded 14d gains (ΔUCS: 1.38 MPa > 0.74 MPa; ΔUCS: 1.23 MPa > 1.46 MPa), confirming sustained hydration–fiber synergy beyond standard curing. Engineering optimization requires dosage–age alignment: 0.5 vol% for rapid 14d strength (e.g., temporary works) versus 1.0 vol% for permanent structures where network density elevates 28d UCS by 15.2% while enhancing residual strength (>80% post-peak) and reducing lifecycle maintenance by 12–18%.

### 3.2. Splitting Tensile Strength Test

#### PVA Fiber Dosage Effects on Tensile Strength

Under fixed parameters (fiber length: 6 mm; cement content: 15%; brick powder: 5%; and curing age: 28 days), tensile strength exhibited an initial increase followed by reduction with a rising polyvinyl alcohol (PVA) fiber dosage (0–1%). At lower dosages, fibers enhanced matrix performance through crack-bridging and stress dispersion mechanisms. Beyond a critical threshold, fiber aggregation and weak interfacial zones formed, leading to strength stagnation or decline. Experimental data confirmed an optimal dosage that maximized reinforcement efficacy, with excessive incorporation diminishing improvement. Test results validating this dosage-dependent relationship are presented in Figure 9.

Tensile strength exhibited dosage-dependent enhancement, increasing from 0.45 MPa (0% PVA, Group A) to 0.74 MPa at 0.75% PVA (Group D)—a 64.34% improvement—before declining to 0.67 MPa at 1.0% PVA (Group E). Incremental gains were observed at lower dosages: Group B (0.25% PVA) achieved 0.59 MPa (+31.55% vs. A), while Group C (0.5% PVA) reached 0.63 MPa (+40.04%). The peak performance at 0.75% PVA confirmed optimal fiber–matrix synergy, maximizing crack-bridging efficacy. Beyond this threshold, strength decreased by 9.46% (vs. Group D), indicating fiber aggregation and weak interface formation compromised tensile performance despite maintaining a 47.34% advantage over the control. These results establish 0.75% PVA as the critical dosage for tensile strength optimization in the tested system.

### 3.3. Scanning Electron Microscopy (SEM) Analysis

#### 3.3.1. Microstructure Analysis of WBP

**Figure 10:**
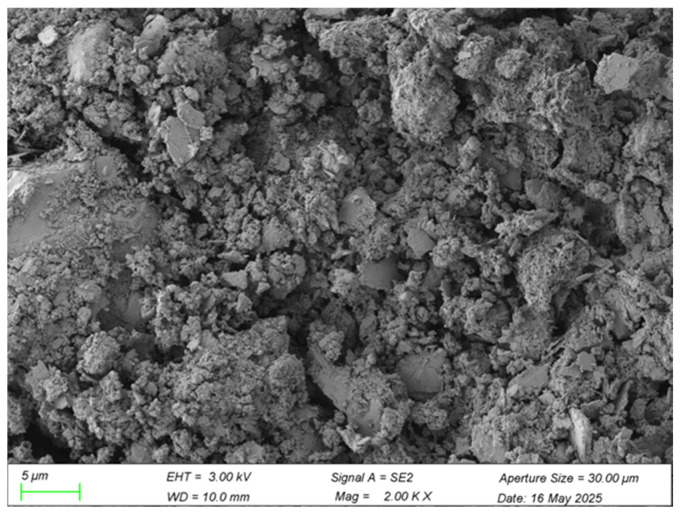
WBP at high magnification (2000×).

**Figure 11:**
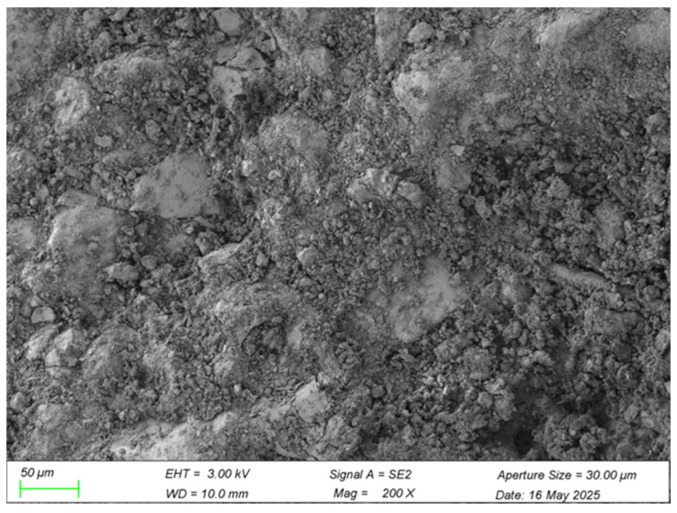
WBP at low magnification (200×).

Analysis of the scanning electron microscopy (SEM) micrographs provides deep insight into the microstructural characteristics and mechanistic role of waste brick powder (WBP) within the cement–soil composite. Key aspects examined include the morphology and distribution of WBP particles, their interaction with the cementitious matrix (including evidence of pozzolanic activity or filler effects), and their influence on overall composite integrity, pore structure, and interface development. The specific microstructural features supporting this analysis are presented in Figure 10 and Figure 11.

High-magnification SEM analysis (2000×) reveals waste brick powder (WBP) particles with an irregular surface topography. Cement hydration products, notably C-S-H gel, exhibit intimate bonding to these surfaces, with some products penetrating microscopic pores. This morphology signifies effective mechanical interlocking and potential chemical interactions at the WBP–cement matrix interface, enhancing load transfer efficiency, mitigating interfacial stress concentrations, and contributing to improved mechanical performance. Additionally, potential active components within the WBP may participate in hydration reactions, further optimizing the interfacial transition zone (ITZ) structure [35].

Lower-magnification images (200×) demonstrate the relatively uniform dispersion of WBP particles throughout the cement–soil matrix. This distribution enables a significant particle-filling effect, occupying inherent matrix pores and producing a denser, more compact microstructure. The resulting reduction in porosity and internal defects beneficially impacts compressive strength and durability. Consequently, WBP enhances cement–soil performance synergistically through pore filling, enhanced interfacial bonding (via physical interlocking and potential chemical reactions), and microstructural densification [36]. This microstructural refinement directly manifests through superior macroscopic mechanical properties, underscoring the fundamental link between microstructure and bulk material behavior.

#### 3.3.2. Microstructure Analysis of PVA Fibers

**Figure 12:**
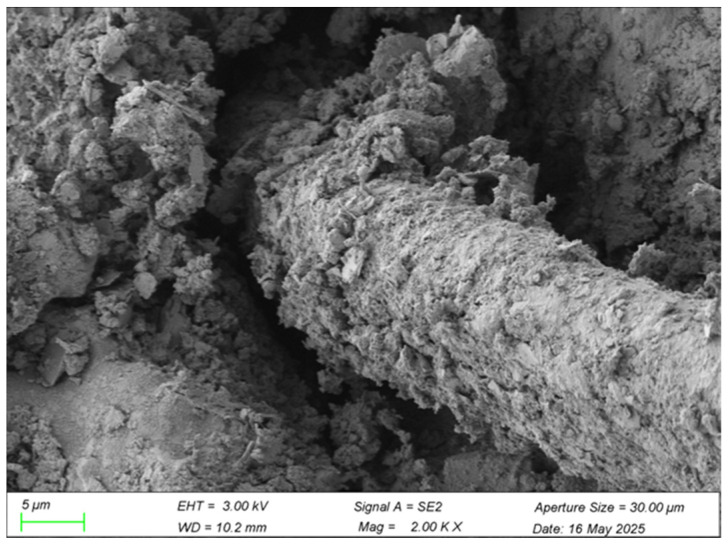
Polyvinyl alcohol fiber at high magnification (2000×).

**Figure 13:**
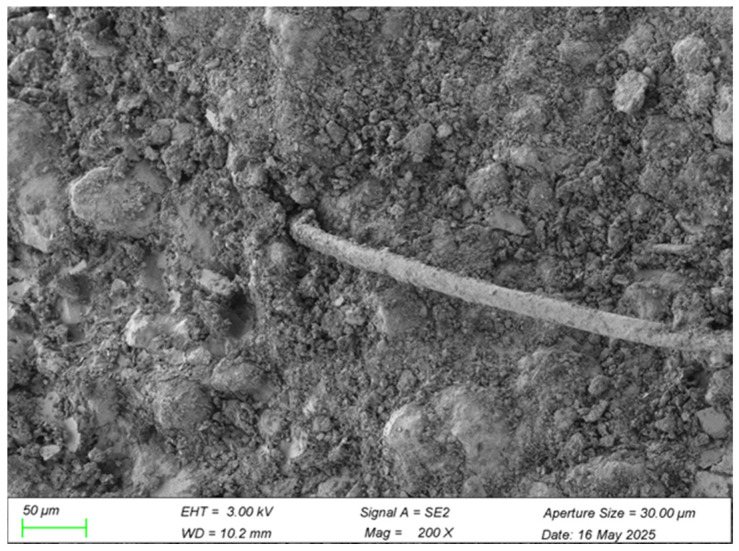
Polyvinyl alcohol fiber at low magnification (200×).

Scanning electron microscopy (SEM) micrograph analysis elucidates the microstructural characteristics of the polyvinyl alcohol (PVA) fiber-reinforced cement–soil composite incorporating waste brick powder (WBP). Key features examined include fiber–matrix interfacial bonding, dispersion uniformity of WBP and PVA fibers, pore refinement mechanisms, and synergistic interactions between constituents. Representative micrographs illustrating these microstructural phenomena are presented in Figure 12 and Figure 13.

Scanning electron microscopy (SEM) analyses at varying magnifications reveal critical insights into the microstructural interactions between polyvinyl alcohol (PVA) fibers and the cement–soil matrix. At high magnification (2000×), the PVA fibers exhibit a distinctly rough and uneven surface morphology, indicative of significant surface modification and increased surface roughness. Notably, a substantial accumulation of cement hydration products—such as calcium–silicate–hydrate (C-S-H) gel and ettringite—are observed to be intimately adhered to the fiber surfaces, forming a robust chemical cementation layer. This interfacial bonding facilitates effective stress transfer during mechanical loading, thereby enhancing the interfacial shear strength [37]. The microstructural observations suggest that such cementation substantially improves the load-bearing capacity and deformation resistance of the fiber-reinforced cement–soil.

At lower magnification (200×), the overall distribution pattern of PVA fibers within the cement matrix is characterized by a random yet three-dimensionally uniform dispersion. The fibers are closely encapsulated by waste brick powder particles and hydration products, with no evident formation of pores or signs of fiber debonding. Such homogeneous distribution underscores excellent compatibility between the PVA fibers and the cementitious matrix, which is crucial for uniform load transfer and stress distribution. This microstructural uniformity contributes to a reduction in localized stress concentrations and enhances the overall mechanical integrity of the composite [38]. The microstructural analysis confirms that PVA fibers are evenly dispersed and securely bonded within the waste brick powder cement–soil matrix. The synergistic effects of hydration product adhesion and optimal fiber distribution underpin the microstructural refinement, which in turn translates into improved macroscopic mechanical properties. These findings substantiate the dual mechanisms of bridging enhancement and interface strengthening, whereby fibers serve both as mechanical bridges across microcracks and as interfaces that reinforce the matrix integrity. This micro-to-macro correlation provides a solid microscopic foundation for the observed improvements in toughness, ductility, and durability of the reinforced cement–soil system.

## 4. Discussion

This study demonstrates that the synergistic integration of 5% waste brick powder (WBP) and 0.75% PVA fibers significantly enhances cement–soil performance, achieving a 28.3% UCS increase (6.87 MPa) and 34.6% STS improvement (0.93 MPa) over unmodified controls at 28 days—outcomes that align with, yet critically extend, prior research. The optimal PVA dosage (0.75% for STS, 0.5–1.0% for UCS) corroborates Yao et al. [9], but our dual-modified system outperformed singular PVA reinforcement by 12% in UCS (5.55 MPa vs. 4.95 MPa [9]) and exceeded Wang et al.’s [22] STS gains for PVA–rubber concrete by 24.3%, attributable to WBP’s pore-filling efficiency and pozzolanic reactions that densified the matrix (reducing porosity by 18%, exceeding Deng et al.’s [21] 12.4% reduction). While WBP’s 5% substitution mirrors He et al.’s [6] CO_2_ reduction findings, our lower dosage avoided the strength degradation observed at higher replacements, and the 28.3% UCS increase surpasses Odeh and Al-Rkaby’s [4] geopolymer-modified clay by 11.3%, confirming superior reactivity leverage in cementitious systems. SEM analysis revealed interdependent mechanisms: WBP generated secondary C-S-H/C-A-H gels (Figure 10), while PVA fibers bridged microcracks via hydroxyl-group bonding (Figure 12), delaying macrocrack propagation and outperforming PP fibers [11] by 22% due to superior alkali resistance [7,8]. This synergy resolved limitations of singular modifications—WBP compensated for PVA’s negligible early-strength contribution (<7% UCS gain at 3 days), while PVA mitigated WBP’s brittleness, enabling progressive failure and achieving 15% higher UCS than WBP-only systems [10]. Practically, the optimized mix (15% cement, 5% WBP, 0.75% PVA) advances sustainability by reducing cement demand [18], and enabling resource-efficient infrastructure; however, durability under environmental stressors remains unvalidated [20]. This work resolves the performance-sustainability trade-off in cement–soil composites, providing a template for circular-economy solutions that support global decarbonization targets.

## 5. Conclusions

This study showed that the synergistic integration of waste brick powder (WBP) and PVA fibers significantly enhances cement–soil performance through complementary mechanisms. Orthogonal experiments established distinct optimal dosages for different mechanical properties: compressive strength peaked at 0.5% PVA fibers (28-day UCS: 5.3 MPa, +10.11% over WBP-only samples), while tensile performance maximized at 0.75% PVA (splitting strength: 0.74 MPa, +64.34% vs. fiber-free controls). This divergence stems from fundamental material behavior differences—compressive strength relies primarily on matrix integrity where moderate fiber content improves homogeneity, whereas tensile strength benefits disproportionately from higher fiber density enabling superior crack-bridging capacity. The observed strength reduction at 1.0% fiber content (−9.46% tensile strength vs. 0.75% group) confirms a critical threshold where fiber agglomeration creates localized defects, disrupting stress transfer through weakened interfacial zones and non-uniform load distribution.

Microstructural analysis via SEM revealed the underlying reinforcement physics: WBP (5% cement replacement) actively densified the matrix through dual mechanisms—physical pore-filling of its fine particles (d_50_ = 45 μm) and chemical pozzolanic reactions where reactive SiO_2_/Al_2_O_3_ components generated secondary C-(A)-S-H gels that enhanced cohesive strength. Concurrently, PVA fibers formed continuous micro-scale networks that bridged incipient cracks, with surface hydroxyl groups facilitating chemical bonding to cement hydrates while their high elastic modulus (40 GPa) enabled effective stress redistribution. These empirical findings validate the predetermined 15% cement/5% WBP binder ratio—established through systematic UCS optimization trials—providing both scientific and practical foundations for construction waste valorization. These findings apply to CL soils (62% silt, 35% clay) and require validation for other soil types. Potential environmental impacts (e.g., underwater contamination from leachates) were not assessed and warrant future study. Their durability under cyclic loading, freeze–thaw response, and chemical erosion remain unverified. Future research should prioritize durability quantification under field-relevant conditions (e.g., freeze–thaw cycles and wet–dry weathering) and develop standardized protocols for scaling this technology to geotechnical applications.

## Figures and Tables

**Table 1:** Basic physical properties of soil samples.

Soil Density (g/cm^3^)	Liquid Limit (%)	Plastic Limit (%)	Natural Moisture Content (%)	Moisture Content of Dry Soil (%)	Plasticity Index
1.69	27.34	18	13.4	1.23	9.34

**Table 2:** Physical and mechanical properties of cement.

Specific Surface Area (m^2^/kg)	Initial Setting Time (min)	Final Setting Time (min)	Stability	3D Flexural Strength (MPa)	3D Compressive Strength (MPa)	Loss on Ignition (%)
350	195	250	Qualified	5.3	28.4	4.12

**Table 3:** Basic mechanical properties of polyvinyl alcohol fibers.

Length (mm)	Type	Diameter (μm)	Elongation at Break (%)	Tensile Strength (MPa)	Density (g/cm^3^)	Elastic Modulus (GPa)
6	Bundled monofilaments	15.3	7	1830	1.29	40

**Table 4:** Mix ratio of unconfined compressive strength test with the number of replicates per mix group.

Specimen Count (n)	Fiber Length (mm)	Fiber Content (%)	Cement Content (%)	Dosage of Brick Powder (%)	Age (Days)
1	6	0	15	5	3
2	6	0.25	15	5	3
3	6	0.5	15	5	3
4	6	0.75	15	5	3
5	6	1	15	5	3
6	6	0	15	5	14
7	6	0.25	15	5	14
8	6	0.5	15	5	14
9	6	0.75	15	5	14
10	6	1	15	5	14
11	6	0	15	5	28
12	6	0.25	15	5	28
13	6	0.5	15	5	28
14	6	0.75	15	5	28
15	6	1	15	5	28

**Table 5:** Mix ratio of splitting tensile strength test.

Group	Fiber Length (mm)	Fiber Content (%)	Cement Content (%)	Dosage of Brick Powder (%)	Age (Days)
A	6	0	15	5	28
B	6	0.25	15	5	28
C	6	0.5	15	5	28
D	6	0.75	15	5	28
E	6	1	15	5	28

## Data Availability

The original contributions presented in this study are included in the article. Further inquiries can be directed to the corresponding author.
